# Evidence-Based Psychological Interventions for the Management of Pediatric Chronic Pain: New Directions in Research and Clinical Practice

**DOI:** 10.3390/children4020009

**Published:** 2017-02-04

**Authors:** Rachael Coakley, Tessa Wihak

**Affiliations:** 1 Division of Pain Medicine, Department of Anesthesiology, Perioperative and Pain Medicine, Boston Children’s Hospital, Pain Treatment Service, 333 Longwood Avenue, Boston, MA 02115, USA; tessa.wihak@childrens.harvard.edu; 2 Department of Psychiatry, Harvard Medical School, 25 Shattuck St, Boston, MA 02115, USA

**Keywords:** chronic pain, pediatric, psychological intervention, parent, child, evidence-based, empirically supported

## Abstract

Over the past 20 years our knowledge about evidence-based psychological interventions for pediatric chronic pain has dramatically increased. Overall, the evidence in support of psychological interventions for pediatric chronic pain is strong, demonstrating positive psychological and behavioral effects for a variety of children with a range of pain conditions. However, wide scale access to effective psychologically-based pain management treatments remains a challenge for many children who suffer with pain. Increasing access to care and reducing persistent biomedical biases that inhibit attainment of psychological services are a central focus of current pain treatment interventions. Additionally, as the number of evidence-based treatments increase, tailoring treatments to a child or family’s particular needs is increasingly possible. This article will (1) discuss the theoretical frameworks as well as the specific psychological skills and strategies that currently hold promise as effective agents of change; (2) review and summarize trends in the development of well-researched outpatient interventions over the past ten years; and (3) discuss future directions for intervention research on pediatric chronic pain.

## 1. Introduction

Current estimates suggest that up to one in four children will have an episode of chronic pain lasting three months or longer [1]. This type of persistent pain is linked to significant physical, psychosocial, and psychological burdens for children and families [2,3]. Chronic pediatric pain also places a significant burden on our healthcare system, ranking among the most expensive pediatric health problems in the United States and costing an estimated $19.5 billion dollars per year [4]. Psychological interventions for pain and pain-related functional deficits (i.e., school impairment, sleep disruption, peer-based challenges, etc.) have been considered an integral part of treatment for the recovery from chronic pain for almost twenty years [5,6]. Taken as a whole, psychological skills and strategies such as cognitive reframing, biobehavioral relaxation, graded in vivo exposure, and mindfulness are demonstrated to decrease pain, improve functioning, increase self-efficacy, and reduce the daily stress that commonly co-occurs in children and adolescents with persistent pain. Moreover, parent-based interventions that reduce maladaptive responses to child pain are demonstrated to further promote a child’s recovery [7]. 

Presently, most psychological interventions for pain are designed with a multi-component structure and are often concerned with evaluating the value-add to standard biomedical approaches to care, the “dose” needed to effect change, the effectiveness of novel delivery methods that can increase access to care, and in many cases assessing how the direct involvement of systems such as parents or families may enhance outcomes. However, the treatment of chronic pain is not a one-size fits all solution and it is unlikely that a single intervention strategy will systematically work for all children with pain. Psychological comorbidities, family-based influences, school environments, developmental factors, functional disability, and underlying pathophysiology of pain all vary by individual. As such, we need to develop various intervention strategies, with the goal of understanding how we can maximally apply these skills and strategies to enhance recovery or coping with pain and to reduce the stress and psychosocial burden for children and parents. 

Despite the proven effectiveness of psychologically based strategies for pain, too few pediatric patients referred to psychological services for pain management obtain services due to a lack of available providers, scheduling conflicts, inadequate knowledge of efficacy, and financial or insurance constraints [8,9]. These logistical barriers highlight the need to create models of care that can be flexible in meeting the time, cost, and limited resource barriers that interfere with service acquisition [9]. Additionally, psychological interventions for pediatric pain must also be packaged and administered in a format that is acceptable, attending closely to the common biomedical biases that may inhibit initial engagement in services. For example, when parents perceive their child to have a solely physical problem, they may dismiss or minimize the need for psychological intervention [8,10,11]. Fortunately, gaining a foundational understanding of the biopsychosocial model of pain helps to illuminate how psychological interventions can effectively foster recovery and may increase the likelihood of obtaining targeted psychological services for pain [12]. 

Helping children and families to effectively manage pain may ultimately be similar to how we help our children succeed in school; all children need to be taught the academic material as well as the skills and strategies that help cement the learning process. How well any individual child succeeds in school, however, is due to a variety of factors such as attentional capacity, intelligence quotient (IQ), motivation, family reinforcement, etc. Children who have poor attention or low motivation, may ultimately be identified as needing additional services such as individual tutoring, small group instruction, or a more structured learning environment to obtain success. Similarly, one of our primary goals in pediatric pain is to identify patient characteristics that may signal the need for more intensive or focused intervention efforts that can help foster recovery [13]. As a corollary, considering that our patients with pain are akin to students in school highlights how first line education and intervention may be necessary, even if not sufficient to produce change for all children. Interventions that provide the building blocks for effective pain management early in a child’s experience of pain—for example, before functional deficits occur—may help to change a child’s long term trajectory in the same way that obtaining a short course of tutoring *before* a child fails a class may ultimately help a child to stay on pace with peers. Thus, in our current line of research and clinical practice we need to think about generating evidence-based first line interventions, such as targeted pain education and cognitive behavioral coping skills, while at the same time devising specialized or more intensive psychological treatments for those with more entrenched difficulties. 

This review includes an overview of pediatric chronic pain intervention research in the past 10 years. Our search terms and findings are presented in Table 1. This is not intended to be an exhaustive list of all interventions tested for all chronic pain conditions, but rather to represent a broad overview of what is being developed and tested for “chronic pediatric pain.” Based on a review of this literature, we will present an overview of current theoretical frameworks that are currently guiding the development of interventions in this field and highlight specific ways in which these frameworks map onto pain-management skills and strategies. We will further evaluate current barriers and challenges in clinical application, and discuss future directions for more seamlessly integrating psychological interventions into the clinical management of pediatric pain.

## 2. Theoretical Foundations

Pediatric pain interventions are based on the understanding that pain is a complex biopsychosocial experience shaped not only by underlying pathophysiology but also by individuals’ thoughts, feelings, and behaviors [43]. Interventions based in the foundational theories of cognitive behavioral, biobehavioral, and acceptance-based models of care currently encompass the majority of psychological interventions for children and adolescents with pain. However, these theoretical approaches are not necessarily mutually exclusive, as many of the current interventions for pediatric pain contain elements of two or more theoretical orientations. Collectively, these theoretical approaches demonstrate promising improvements in self-efficacy, self-management, family functioning, psychosocial well-being, pain severity, school attendance, anxiety, depression and feelings of hopefulness [7,44,45]. The vast majority of evidenced-based interventions for pediatric pain are designed as brief, goal-oriented treatments incorporating learning experiences that help to modify negative cognitions about pain and eliminating dysfunctional behavioral patterns that occur as a result of the pain. Psychological interventions for pain are also commonly designed to help un-link the association between the sensory experience of pain and the cognitive affective evaluation of the pain that triggers a fight or flight response, often referred to as a “pain alarm” [46]. Specifically, most psychological treatments for pain are designed to address the negative or fearful thoughts about pain (e.g., ‘it’s going to hurt too much’, ‘I can’t do this’) that produce physiological changes (e.g., racing heart, shortness of breath, increased discomfort) and resultant behavioral changes (e.g., withdrawal from activity, poor sleep). Interventions may also use strategies such as graded exposure to anxiety producing stimuli and reinforcement for adaptive behaviors. 

As pediatric pain is conceptualized as a biopsychosocial process, intervention efforts are ideally targeted towards a systems framework inclusive of parents, families and schools. It has been well documented that the way parents respond to their child’s pain is associated with pain severity, functional disability and other somatic complaints [3,7,47]. Moreover, without targeted parent support at home and school, it is more challenging for children and adolescents to make steady gains. Parents can be collaborators in treatment when they are involved in the planning and reinforcement of their child’s psychological intervention plan [48]. For example, parents may be taught how to use relaxation techniques to help reinforce the use of these strategies at home, or may collaborate by consistent provision of rewards or consequences for a child’s established behavior plan [17]. But perhaps more importantly, parents can be co-treated along with their child. For example, it’s common for parents to become highly focused on their child’s pain. This attention to pain is known to inadvertently reinforce a child’s focus on his or her own pain increasing disability [49,50]. In contrast, when parents are taught how to reduce their focus on a child’s pain, a child’s pain and function may improve [27]. Research also suggests that parent-focused treatments such as Problem Solving Therapy (PST) may directly improve parent behaviour and parent psychological functioning [51], an important outcome given that parents are known to experience significant stress in the context of their child’s pain [52]. 

School performance has also been an identified target for intervention because functional impairment in schools—including poor grades, absenteeism, and peer victimization—is a well-documented problem for children with pain [53,54,55]. It’s easy to understand why pain can reduce a child’s ability to cope with school demands and contribute to poorer grades. However, for some children school pressures and peer victimization may also be a contributory factor to the onset of pain, thus requiring intervention to reduce the underlying psychosocial stress.

## 3. Intervention Components

Psychologists typically teach a broad complement of cognitive, behavioral, and biobehavioral strategies, often in the context of multidisciplinary treatments that might also incorporate physical therapy and non-opioid medications. The literature includes many reports of effective multicomponent Cognitive Behavioral Therapy (CBT) treatments for pediatric chronic pain (see Table 1). However, with some notable exceptions for conditions such as headache pain, [5] there is little conclusive evidence about which component parts of psychological intervention are most effective for which population of children with pain. Many investigations have tried narrowing down this area of research by investigating interventions via defined primary pain (e.g., abdominal, musculoskeletal, neuropathic), developmental level (children vs. adolescents), pain severity, psychological comorbidities (e.g., anxiety, depression) and other factors. Yet, in clinical practice, where the majority of children with chronic pain have more than one pain condition and an array of psychological and psychosocial stressors [1], these research-defined classifications may be less salient. 

While randomized controlled trials (RCTs), both individually and collectively evaluated in meta analyses, provide our best evidence that psychological strategies offer benefit to pediatric patients with pain, a broader examination of the current research in this field may help us to better conceptualize new directions in this field. Specifically, we can learn how evidenced-based psychological interventions are being implemented across pediatric pain conditions, in various settings, using novel delivery methods and unique combinations of skills and strategies. 

Below we briefly review the most common outpatient intervention frameworks cited in the last ten years (2005–2015), highlighting how a variety of skills and strategies may be applied in helping children, adolescents, and parents in the management of pediatric pain. 

### 3.1. Pain Education

Psychoeducation is a fundamental component of most interventions for pediatric pain. Educational interventions provide a rationale for how and why psychological strategies can effectively reduce pain and pain-related stress and improve daily function. The type of psychoeducation that is empirically shown to improve chronic pain outcomes has been termed “Neuroscience Education (NE).” NE is described as an educational intervention that clearly explains “the neurobiology and neurophysiology of pain and pain processing in the nervous system” [56]. The goal of NE is to teach patients about the basic neuroscience of pain—for example, nerve hypersensitivity, central sensitization, neuroplasticity, and brain modulation of pain signals—to enhance a patient’s understanding that pain experience does not necessarily represent ongoing harm to the body. Research suggests that thinking about pain in this way can produce immediate and long term improvement in pain severity, physical activity, fear, and catastrophic thinking [57,58,59]. Additionally, NE can provide clear evidence regarding the bi-directional relationship between stress and pain and a rationale for how and why cognitive-behavioural strategies can effectively reduce pain and restore function. NE has been well studied in adults with chronic pain [56], but to date no pediatric pain investigations have examined an exclusive NE approach. 

### 3.2. Cognitive Reframing/Positive Self-Statements

Cognitive reframing refers to modifying one’s thoughts about a distressing situation so that they are less focused on negative aspects of the situation. Typically, children are taught first to *identify* their negative thoughts or ‘self-talk’ about pain, then to *challenge* these thoughts, for example, by listing out evidence in support of the thought and evidence against it, and finally to *modify* these thoughts to produce less negative emotional responses. A related technique, positive self-statements, focuses on developing a set of statements that emphasize one’s ability to cope positively with a challenging situation. These techniques are widely applied, as persistent negative thinking, referred to as ‘catastrophizing’, is commonly associated with chronic pain [50,60,61,62] and can contribute to increased pain and disability [63,64]. Modifying catastrophic thinking—for children with pain and their parents—can help to foster adaptive recovery [51].

### 3.3. Graded Exposure and Psychological Desensitization

Exposure and psychological desensitization techniques involve gradually increasing exposure to a feared stimulus over time while minimizing associated anxiety. Typically, systematic desensitization entails developing a hierarchy of anxiety-provoking situations ordered from least- to most-feared. The goal is to encounter the situations safely, without the previously experienced distress, to unlink them from associated anxiety. The clinician may teach the child to engage in positive coping strategies (e.g., relaxation) during this desensitization process. Desensitization can also be used to reduce this pain-avoidance pattern, decrease fear of pain, and help children to re-engage in activities, which can in turn help to reduce pain [24,65]. 

### 3.4. Biobehavioral Relaxation Techniques

Relaxation is widely used in the treatment of chronic pain. Relaxation strategies focus on acquiring specific skills that can be used to decrease pain perception by promoting a general sense of physical and psychological well-being. The decreased physiological state of arousal that results from the practice of different relaxation based strategies may also serve to diminish pain and its related emotional symptoms. Relaxation approaches include diaphragmatic breathing, progressive muscle relaxation (PMR), and guided imagery techniques. Hypnosis also relies heavily on relaxation and has demonstrated benefit, especially for youth with IBS or functional abdominal pain disorders [66,21]. In a meta-analysis published in 2010, Palermo and colleagues concluded that relaxation asa stand-alone cognitive-behavioural treatment was effective in reducing pain across studies of children and adolescents with a variety of chronic pain conditions including headache, abdominal pain, and fibromyalgia [45]. Typically, however, relaxation strategies are incorporated into multicomponent interventions.

### 3.5. Biofeedback/Biofeedback-Assisted Relaxation Training (BART)

Biofeedback is a therapeutic intervention that measures and ‘feeds back’ information about an individual’s physiological activity (typically indicators of autonomic arousal) to increase awareness of and control over one’s physiological processes. Biofeedback for pain management typically incorporates a focus on respiration (pace and depth of breathing), muscle electromyography, peripheral skin temperature, and blood flow or heart rate variability. The ‘feedback’ can be visual and/or auditory. Children often find biofeedback engaging and benefit from the concrete, visual aspects of this technique. While biofeedback is shown to have positive effects on pain severity [45], additional well-designed studies are needed to compare biofeedback training to placebo conditions to determine whether there is a potential influence of the novelty of this treatment approach. 

### 3.6. Acceptance-Based Approaches 

There is growing evidence that the cognitive practice of acceptance and “staying-in the-moment” combined with the behavioral approach-based practice of actively engaging in valued activities may be an important component of treatment for chronic pain. Research on these strategies demonstrates their effectiveness in adult chronic pain populations [67,68]. At this time, there is a limited but growing body of literature supporting the application of these skills in children with chronic pain [69]. 

### 3.7. Other

A few other treatment modalities including techniques from structural family therapy, interpersonal therapy [17], coping skills training [14], social learning [37] and problem solving therapy [70] have been used in the treatment of chronic pain. These have had fewer completed investigations, but suggest that there may be additional interventions that may offer benefit. 

## 4. New Developments in Evidence-Based Research

### 4.1. Primary Pain

The first generation of pain treatment interventions almost exclusively focused on diagnostic-specific populations (e.g., gastrointestinal pain, headache, complex regional pain syndrome. Developing interventions that are specific to a presenting pain problem has helped to establish an important foundation in pain management, ,but in some cases this diagnostic specific focus may unintentionally obscure opportunities to more broadly address the psychological challenges that universally impact recovery from chronic pain [71]. In contrast, treating chronic pain as a primary disorder “promotes the conceptualization of chronic pain as a more generalized nociceptive process with common functional limitations (e.g., sleep, school function, activity) and emotional correlates (e.g., fear, worry, hopelessness, helplessness)” [72]. Additionally, a primary pain conceptualization opens the door for the functional treatment of pain even when a clear biomedical etiology is not determinable. From an intervention standpoint, conceptualizing pain as a primary disorder means that interventions have the potential to serve a wide population base, inclusive of various pain disorders. In the research reviewed, about 30% of the studies included multiple pain populations, suggesting that viewing pain as a primary disorder may be a viable way to design and evaluate intervention efficacy. For patients with more entrenched psychological or functional deficits, more comprehensive interventions may be most effective when they specifically target psychological symptoms—for example depression, anxiety, avoidance behaviors, family factors, or fear of pain—as opposed to pain-specific etiologies [71]. 

### 4.2. Evaluating Intervention Delivery 

With a growing evidence base demonstrating short and long-term benefits for psychological intervention for pain, developing effective methods for disseminating these skills and interventions to the millions of children and parents who need them most is part of the new frontier for this line of research. The historical model of providing 1:1 individualized psychological intervention may not be the most efficient model of care for pediatric pain for several reasons. First, as chronic pain is a relatively new area of practice in pediatric psychology, there are too few pediatric psychologists to meet the demand for intervention. In urban areas, this deficit can contribute to lengthy wait lists for services and in rural areas there may not be any psychologists available to provide this relatively specialized area of treatment. Second, while biomedical biases may continue to inhibit engagement in traditional psychotherapy interventions, novel delivery methods that frame psychological intervention as a component part of pain treatment or as a way to attain skills or support for a challenging problem, may help to reduce this bias and increase patients’ view of the intervention as an integrated part of their care. Third, traditional models of psychotherapeutic intervention commonly require weekly or bi-weekly in-person visits. Chronic pain treatment can often be very costly and demanding on a family’s schedule requiring frequent appointments such as physical therapy, occupational therapy, tutoring, and physician visits. Developing psychological intervention platforms that can effectively deliver evidence-based treatment in cost, time and resource efficient ways may enhance patient engagement and outcomes. 

#### 4.2.1. Internet and Telehealth

There has been a recent emergence of Internet-based CBT interventions for pediatric pain [73]. Internet-delivered CBT for chronic pain has a number of advantages to traditional treatment delivery models. It reduces waiting lists and scheduling conflicts, can be self-paced, and minimizes stigma [74,75]. Moreover, while time-intensive and costly to develop, web-based programs can be extremely cost and time efficient from a patient care perspective. Web-based protocols have been found to be a promising way to introduce pain education, thought restructuring, relaxation training, and other CBT skills. Additionally, some programs can teach parent-based strategies to reinforce positive coping and improve parent-child communications [22]. Findings of a RCT demonstrate significant reductions in pain and activity limitations post-treatment, with gains maintained at a 3-month follow-up. Several Internet-based CBT interventions for children with headaches have also demonstrated reductions in pain and in pain-related negative thinking [19,30].

Limitations to internet delivery methods include the automated aspects of this approach, requirement of access to appropriate technology, and in the case of unguided approaches, a lack of immediate response to individual needs [75]. Moreover, while some interventions have shown benefit [76,77], other studies have high attrition rates suggesting it can be challenging to engage children and adolescents via a home-based, self-administered program [19]. 

Telehealth treatments that can be personalized and delivered into a patient’s home via face-to-face software programs (e.g., skype), personalized email or individualized phone contacts, has also received increased attention in the pediatric pain research over the past 10 years. In most cases, the telehealth portion of an intervention was designed to reinforce skills that were previously taught via other modalities and to problem solve new situations. As intervention dissemination continues to expand, additional telehealth, eHealth and mHealth technologies will likely become more available. Whether these interventions will be sufficient as stand-alone treatments or will alternatively become a valued adjunct to in-person therapies may also be a focus of future research. 

#### 4.2.2. Group Interventions 

Group-based CBT interventions have been shown to be cost- and time-efficient and as effective as individual treatment in numerous studies [78,28]. The group format also promotes psychosocial support, a particularly salient factor for children with chronic pain who often feel alienated by their condition [79]. Group therapy programs can also incorporate other family members in treatment sessions to help modify pain responses within the family environment [40,80]. In the pediatric literature, group CBT has already demonstrated effectiveness for a variety of pain-related problems such as irritable bowel syndrome [15], headache [26] and pain-related school absenteeism [28]. The most common limitations to group treatments include the possibility of missing sessions and therefore only attaining a partial dose of the interventions. Relatedly, all of the group treatments in this review were administered in person. Thus, access to group treatment may be a logistical barrier.

### 4.3. Inclusion of Psychosocial Systems

Overwhelmingly, the research on pediatric pain in the past ten years has incorporated parents into treatment. This is an excellent example of how the foundation of our evidence- based research has informed current practice. With strong evidence finding that for all psychological interventions in pediatric pain, inclusion of parents in the treatment fosters better outcomes, it’s encouraging to see that the vast majority of newly designed interventions are focused on treating parents. Though almost 70% of the studies we reviewed included parents in their target population, there was tremendous variability in how parents were integrated into treatment. For example, some studies provided only a session or partial session of treatment to parents [17], while other studies fully incorporated parents into all aspects of the treatment [14]. 

Despite our understanding that other psychosocial systems such as family environments [51,52,81,82] and schools [55,83,84,85] can influence a child’s pain and recovery, there remain few interventions designed to address these broader systems. In our current review, only a single study included siblings, parents, and peers [40] and no studies directly targeted schools. Part of the challenge may be that these more contextual environments require more comprehensive models of care that may be considered too logistically challenging or costly for wider scale clinical implementation. However, given that chronic pain is among the most common and costly problems for children, evaluating whether or not targeted modification of these systems may provide significant benefit may ultimately offer cost benefit. 

## 5. Future Directions

Over the last ten years, our increased knowledge of the neurobiology of pain in combination with a promising research foundation demonstrating that evidenced-based psychological intervention can offer immediate and long-term benefit to children with chronic pain, has led to more nuanced questions regarding the dose, application and dissemination of these interventions. For example, the majority of interventions have been designed as brief models of care (<8 h), increasing our confidence that positive outcomes are possible within a relatively short duration and enhancing opportunities to treat more patients. Effective group treatments similarly enhance opportunities to treat larger numbers of patients while simultaneously providing social support. Moreover, new models of care including internet and telehealth assisted delivery, suggest that our current technology affords opportunities to bring these evidence-based skills and strategies into patients’ homes, eliminating many of the logistical barriers to treatment. 

Even with these advances, intervention research for pediatric chronic pain is still in its relative infancy and there remain several gaps in our intervention research. For example, our review finds that many interventions did not clearly specify the duration of treatment, leaving out important details such as the number of sessions or the length of each session. Without this “dose” information, it can be difficult to compare efficacy across interventions and challenging to determine how much treatment might be needed for any particular child or family. The use of a tiered care approach to treatment, whereby various levels of intervention intensity are recommended based on a child’s particular profile, may emerge as a useful way to direct care. Thus, the availability of various “doses” of intervention is highly relevant. In some cases, patients may first be directed through lower levels of intervention with non-responders proceeding to more intensive interventions, though the use of rapid screening tools (e.g., Pediatric Pain Screening Tool [86]) may also allow providers to identify patients who, according to identified risk factors (i.e., functional disability, depression) need to be fast-tracked to higher levels of intervention (e.g., Intensive Interdisciplinary Rehabilitation Therapy).

Within the current model of care there still remain too many patients who do not seek or attain any psychological interventions for pain due to biomedical biases [8]. Continuing efforts to disseminate basic neuro-science education and to arm children and parents with the necessary skills and strategies early in a child’s experience of chronic pain, or even *preventively*, remains a goal for intervention research. Interventions that use non-diagnostic titles such as “The Comfort Ability” [80], “Web-Map” [76], “Teens Taking Charge” [77] or “Headstrong” [16] may help to reduce patient bias [87,88]. Additionally, including psychological interventions as an integrated part of an interdisciplinary treatment program can enhance acceptability. Recent success with social media efforts to shift public opinion about the management of acute pain in children [89] suggests that beyond our focus on patient bias, pediatric psychologists may also have a role to play in shifting general public awareness towards a broader understanding of psychological intervention for managing pediatric chronic pain. 

When patients do demonstrate a willingness to engage in psychological intervention for chronic pain, we still have too few options available for clinical use. To date, even with the proliferation of research-based internet programs, there are no web-based protocols available for public use. Moreover, only a handful of the interventions reviewed over the past ten years are maintained outside the research arena as ongoing clinical services. Thus, we need to continue to focus efforts on how to effectively share our knowledge and integrate evidence-based interventions into our current healthcare systems. Programs such as The Comfort Ability, a one-day CBT workshop for children with pain and their parents, is one such example. This program, inclusive of leaders’ manuals and patient and parents’ workbooks, is designed as a first-line intervention for pediatric pain. Now actively running at eight healthcare institutions, it has demonstrated feasibility for transfer to other sites. 

While evidence in the field suggests that many patients and parents benefit from first-line education and intervention, there is also a need to become more specialized in our approach to intervention for those who have high levels of disability or complex comorbidities. There is a need to more fully establish a personalized medicine approach, enabling the identification of patient-specific mood, cognitive, developmental and behavioral patterns that may map onto specialized or tailored interventions leading to improved long-term pain-related outcomes. Without such efforts, patients may become frustrated with trial and error approach or even become treatment resistant [71]. One such example of a specialized approach to care is the “GET Living” intervention, designed to target pain-related fear [90]. With past research demonstrating that patients with high pain-related fear may only partially respond to general CBT [91], more tailored or specialized treatment is warranted for this patient subgroup. Graded in vivo exposure, a specific cognitive–behavioral technique that is effective at improving function for individuals with pain-related fear is being evaluated as the hallmark treatment of GET Living. Though this more intensive treatment is not needed for all pediatric patients with pain, the specialization of this intervention may provide an important path forward for patients with pain and high levels of associated fear. 

## 6. Conclusions

In conclusion, pediatric chronic pain intervention research in the past ten years has made tremendous gains. Collectively there is compelling research demonstrating meaningful gains in reducing pain severity and improving functional outcomes for many brief, outpatient interventions. Nevertheless, there remains a continued need to develop interventions that help to diminish biomedical biases and reduce logistical barriers to care. Moreover, we need sustained efforts in identifying and developing first-line interventions that have wide-scale applicability as well as more nuanced and targeted treatment for patients with more psychophysiological complexities. Psychologists must also consider how to translate our current research initiatives into routine practice for children with chronic pain. Beyond developing interventions, we must continue to educate patients, providers and the general public toward a comprehensive understanding of how and why these interventions can foster adaptive recovery. As our knowledge of the neurobiology of pain continues to expand and we continue to gain confidence in our intervention research, it will be increasingly possible to effectively implement and facilitate the adoption of our evidence-based research. 

## Figures and Tables

**Table 1:** Evidence-Based Psychological Interventions for Pediatric Chronic Pain from 2005–2015.

Authors, Year	Target Population ^1^	*N*	Pain Type ^2^	Therapy Type ^3^	Duration: Session/Weeks (*Total Time*)	Mode of Delivery	Setting	Outcome
Kashikar-Zuck et al., 2005 [14]	Adol Parent	30	MSK	CST	4 weeks individual/parent-adol + 2 biweekly telehealth (*unknown*)	Individual + 3 parent-adol sessions + telehealth	Outpt	Reduced functional disability and depressive symptoms in CST group and self-monitoring group at post-treatment. CST showed greater improvement in coping skills and trend towards reduced pain intensity.
Robins et al., 2005 [15]	Child Adol Parent	69	AB	CBT	5 weeks (*3–4 h*)	Family-based	Outpt	Reduced pain intensity compared to standard medical care alone at post-treatment and 1-year follow-up.
Connelly et al., 2006 [16]	Child Adol	37	HA	CBT	4 weeks (*4 h*)	CD-ROM + telehealth	Home	Reduced pain intensity, frequency and duration compared to standard medical care waitlist control at 1-, 2- and 3-month follow-up.
Degotardi et al., 2006 [17]	Child Adol Parent	67	MSK	CBT, +SFT + IP	8 weeks (*unknown*)	Parent-child/adol group + weekly parent meetings	Outpt	Reduced pain intensity, functional disability, somatic symptoms, anxiety and fatigue at post-treatment.
Duarte et al., 2006 [18]	Child Adol Parent	32	AB	CBT	12 weeks (*3–4 h*)	Family-based	Outpt	Reduced frequency of pain crises compare to standard medical care at post-treatment.
Hicks et al., 2006 [19]	Child Adol Parent	47	Multi	CBT	7 weeks (*unknown*)	Internet + telehealth	Home	Reduced pain intensity compared to standard medical care waitlist control at 1 and 3-mo follow-up.
Abram et al., 2007 [20]	Child Adol Parent	81	HA	CBT + ED	1 day *(1 h)*	Parent-child/adol group	Outpt	Increased headache knowledge and reduced physician face-to-face time compared to neurological consultation group at 3 and 6months post-treatment. Reduced headache-related disability in both groups.
Vlieger et al., 2007 [21]	Child Adol	53	AB	HT	12 weeks (*5 h*)	Individual	Outpt	Reduced pain intensity and frequency compared to standard medical care at 1-year follow-up.
Palermo et al., 2009 [22]	Adol Parent	48	Multi	CBT	8 weeks (*4 h child + 4 h parent + 1 h therapistcontact*)	Internet + telehealth	Home	Reduced pain intensity and functional disability compared to standard medical care wait-list control at post-treatment and 3-month follow-up.
van Tilburg et al., 2009 [23]	Child Adol Parent	34	AB	Guided Imagery	8 weeks *(2–3 h)*	Portable CD Audio-Recordings	Home	Reduced pain intensity, functional disability and improved QOL for audio exercises compared to standard medical care alone at post-treatment and 6-month follow-up.
Wicksell et al., 2009 [24]	Child Adol Parent	32	Multi	ACT	10 weeks *(4.5 h individual + 1.5 h parent-child/adol)*	Individual + 1-2 parent-child/adol sessions	Outpt	Reduced pain intensity, functional disability, pain intensity and pain-related worry compared to MDT group at post-treatment and at 3.5- and 6.5- month follow-up.
Barakat et al., 2010 [25]	Adol Parent	53	SCD	CBT	3 weeks *(4–5 h)* + 1 booster *(1.5 h)*	Family-based	Home	Exploratory analyses showed small to medium effects in favor of CBT group on pain frequency, health service use, SCD knowledge, and family cohesion at post-treatment.
Gerber et al., 2010 [26]	Child Adol Parent	34	HA	SCT + SMT + PCST	8 child sessions *(12 h)* + 4 parent sessions *(8 h)*	Child group + parent group	Outpt	Reduced headache frequency and duration and improved school and daily functioning in multimodal behavioral education group and BFT group at post-treatment.
Levy et al., 2010 [27]	Adol Parent	200	AB	SLCBT	3 weeks *(3 h)*	Family-based	Outpt or Home	Reduced pain, gastrointestinal symptom severity and parental solicitous responses to child symptoms compared to educational intervention at post-treatment and 1-week, 1- and 3-month follow-up.
Logan and Simons, 2010 [28]	Adol Parent	40	Multi	CBT	4 weeks or day workshop *(4 h adol + 4 h parent-adol)*	Adol group + parent-adol group	Outpt	Reduced pain intensity, negative mood/self-esteem and improved school functioning at post-treatment.
Stinson et al., 2010 [29]	Adol Parent	46	MSK	CBT + ED	12 weeks *(5 h)*	Internet + telehealth	Home	Improved JIA-related knowledge and average weekly pain intensity compared to internet intervention control group at post-treatment.
Trautmann and Kroner-Herwig, 2010 [30]	Child Adol	65	HA	CBT vs. Self-Help/ RT	6 weeks *(unknown)*	Internet + telehealth	Home	Reduced pain frequency, duration and catastrophizing in CBT, AR and educational intervention groups at post-treatment.
Warner et al., 2011 [31]	Child Adol Parent	40	Multi	CBT	10 weeks *(9–12 h child + 2 h parent)* + 2 booster	Individual + 3 parent meetings	Outpt	Reduced anxiety and somatic symptoms compared to standard medical care waitlist control at post-treatment and 3-month follow-up.
Kashikar-Zuck et al., 2012 [32]	Child Adol Parent	114	MSK	CBT	8 sessions *(6 h child + 2–3 h parent-child/adol)* + 2 boosters *(1.5 h)*	Individual + 3 parent-child/adol sessions	Outpt	Reduced pain intensity, functional disability and depressive symptoms in CBT group and Fibromyalgia Education group at post-treatment. CBT showed greater reduction in functional disability compared to fibromyalgia education.
Law et al., 2012 [33]	Adol Parent	26	Multi	CBT	17–27 weeks *(unknown)*	Internet + telehealth	Home + Outpt	Sending messages to online coach was associated with reduced pain intensity and functional disability at post-treatment.
Myrvik et al., 2012 [34]	Child Adol Parent	10	SCD	BFT + RT	1 day *(1 h)*	Parent-child/adol + telehealth	Home + Outpt	Reduced pain frequency at post-treatment and 6-week follow-up.
Vlieger et al. 2012 [35]	Child Adol	52	AB	HT	12 weeks *(5 h)*	Individual	Outpt	Reduced pain, pain frequency and somatic symptoms at mean follow-up of 4.8 years.
Kashikar-Zuck et al., 2013 [36]	Adol Parent	114	MSK	CBT	9 weeks *(unknown)*	Individual + 3 parent-adol sessions	Home	Improved functional disability at post-treatment.
Levy et al., 2013 [37]	Child Parent	200	AB	SLBT	3 weeks *(3 h)*	Family-based	Outpt or Home	Reduced pain and improved coping skills at 1-year follow-up compared to education group. Similarly, SLCBT group exhibited decreased parental solicitousness and maladaptive pain-related beliefs.
Shiri et al., 2013 [38]	Child Adol	10	HA	BFT	10 sessions *(5 h)*	Individual virtual reality	Outpt	Reduced pain and improved QOL and daily functioning at 1 and 3 months post-treatment.
Stern et al., 2014 [39]	Child Adol	27	AB	BFT	8 sessions *(4 h)*	Individual	Outpt	Reduced pain frequency and severity at post-treatment and 2-week follow-up.
Armbrust et al., 2015 [40]	Child Adol Parent Sibling	64	MSK	CBT+ ED	14 weeks *(14 h)*	Internet + 4 group with parent + 1 group with sibling/friend	Outpt	Program commitment similar to internet-based JIA self-help program via phone support and higher commitment compared to other internet interventions for youth.
Hesse et al., 2015 [41]	Adol	20	HA	MBI	8 weeks *(16 h)*	Group	Outpt + Home	Adolescents report improved depressive symptoms and pain-related acceptance at post-treatment. Parents report improved QOL and physical functioning.
Law et al., 2015 [42]	Adol Parent	83	HA	CBT	8–10 weeks *(4 h adol + 4 h parent + 1 h online coach)*	Internet + telehealth	Outpt +Home	Reduced headache frequency in Internet CBT group and headache treatment group at post-treatment and 3-month follow-up.

**Search Terms:** pediatric/child/adolescent chronic pain + intervention; CBT; biobehavioral; cognitive behavioral; education; psychoeducation; parent training; hypnotherapy; pain coping; mindfulness; acceptance; internet; telehealth; group. **^1^ Target Population:** Children = ages 7–11, Adolescents (Adol) = ages 12–18. **^2^ Pain Type: ** MSK = musculoskeletal, HA = headache, AB = abdominal, Multi = multiple pain, SCD = sickle cell disease, Neuro = neuropathic. **^3^ Therapy Type:** ACT = Acceptance and Commitment Therapy, BFT = Biofeedback Therapy, CBT = Cognitive Behavioral Therapy, ED = Psychoed CST = Coping Skills Training, HT = Hypnotherapy, IP = Interpersonal Therapy, MBI = Mindfulness-Based Intervention, MDT = Multidisciplinary Treatment, PCST = Pain Coping Skills Training, RT = Relaxation Therapy, SCT = Sensory Coping Training, SFT = Strategic Family Therapy, SLCBT = Social Learning and Cognitive Behavioral Therapy. ^4^ **Setting:** Outpt = Outpatient.
